# Supplementary material for: Identification of photoperiod-induced specific miRNAs in the adrenal glands of Sunite sheep (Ovis aries)
Source: Front Vet Sci. 2022 Jul 22;9:888207. doi: 10.3389/fvets.2022.888207 (PMC9354845; doi:10.3389/fvets.2022.888207)
Supplement: Supplementary file 1 [file Data_Sheet_1.ZIP › Supplementary file/miRDeep2 file.zip!/miRDeep2 file/Know_Structure/LP42A3/oar-mir-377.pdf]

miRBase precursor : oar-mir-377
Total read count : 74
oar-miR-377-5p read count : 23
oar-miR-377-3p read count : 37
remaining reads : 14

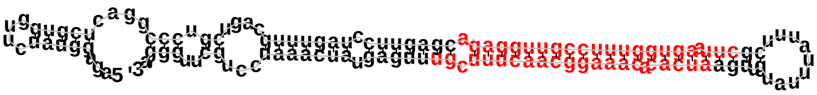

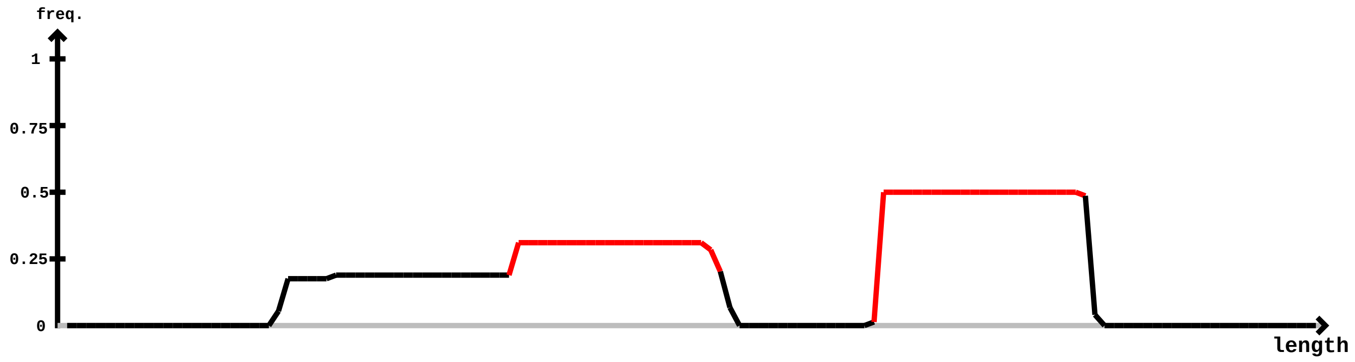


```
                                                                                                    oar-miR-377-3p
                                              oar-miR-377-5p
5'- agagguaucuuggugcucaggcccugcugacguuugauccuugagcagagguugccuuuggugaauucgcuuuauuuauguugaaucacacaaaggcaacuuucguuugaguaucaaauccugcuuggga  -3'  exp
    ...(((((....)))))....(((.((....(((((((.(((((((.(((((((((((((((((.(((((.........).)))))))).))))))))))))).))))))).)))))))...))..))).   reads  mm
    .....................cccugcugacguuugauccuugagc....................................................................................   4      0
    ......................ccugcugacguuugauccuugagc....................................................................................   9      0
    ...........................ugacguuugauccuugagc....................................................................................   1      0
    ..............................................agagguugccuuuggugaau................................................................   2      0
    ..............................................agagguugccuuuggugaauu...............................................................   6      0
    ..............................................agagguugccuuuggugaauuc..............................................................   10     0
    ..............................................agagguugccuuuggugaauucg.............................................................   5      0
    ...................................................................................aaucacacaaaggcaacuuucgu........................   1      0
    ....................................................................................aucacacaaaggcaacuuucg.........................   1      0
    ....................................................................................aucacacaaaggcaacuuucgu........................   32     0
    ....................................................................................aucacacaaaggcaacuuucguu.......................   3      0
```
